# Hypoxic Transformation of Immune Cell Metabolism Within the Microenvironment of Oral Cancers

**DOI:** 10.3389/froh.2020.585710

**Published:** 2020-12-16

**Authors:** Amrita Chaudhary, Swarnendu Bag, Neeraj Arora, Vivek S. Radhakrishnan, Deepak Mishra, Geetashree Mukherjee

**Affiliations:** ^1^Department of Histopathology, Tata Medical Center, Kolkata, India; ^2^Department of Laboratory Hematology and Molecular Genetics, Tata Medical Center, Kolkata, India; ^3^Department of Clinical Hematology, Tata Medical Centre, Kolkata, India

**Keywords:** hypoxia, immune cells, metabolism, microenvironment, oral cancer

## Abstract

Oral squamous cell carcinoma (OSCC) includes tumors of the lips, tongue, gingivobuccal complex, and floor of the mouth. Prognosis for OSCC is highly heterogeneous, with overall 5-year survival of ~50%, but median survival of just 8–10 months for patients with locoregional recurrence or metastatic disease. A key feature of OSCC is microenvironmental oxygen depletion due to rapid growth of constituent tumor cells, which triggers hypoxia-associated signaling events and metabolic adaptations that influence subsequent tumor progression. Better understanding of leukocyte responses to tissue hypoxia and onco-metabolite expression under low-oxygen conditions will therefore be essential to develop more effective methods of diagnosing and treating patients with OSCC. This review assesses recent literature on metabolic reprogramming, redox homeostasis, and associated signaling pathways that mediate crosstalk of OSCC with immune cells in the hypoxic tumor microenvironment. The likely functional consequences of this metabolic interface between oxygen-starved OSCC and infiltrating leukocytes are also discussed. The hypoxic microenvironment of OSCC modifies redox signaling and alters the metabolic profile of tumor-infiltrating immune cells. Improved understanding of heterotypic interactions between host leukocytes, tumor cells, and hypoxia-induced onco-metabolites will inform the development of novel theranostic strategies for OSCC.

## Introduction

Squamous cell carcinoma (SCC) accounts for more than 95% of all cancers affecting the head and neck region, with high rates of associated mortality and morbidity that represent a major public health burden worldwide [1, 2]. In oral squamous cell carcinoma (OSCC), the predominant sites involved include the tongue and the gingivobuccal complex, and mortality rates can be substantially reduced by early detection and prevention strategies [3]. However, despite the development of several high-throughput multimodal diagnostic tools, early stage detection is still problematic; hence, 5-year survival rates in recurrent and metastatic disease remain extremely poor [4]. It remains unclear to what extent radiotherapy or chemotherapy exerts stimulatory or suppressive effects on host leukocyte responses [5]. The immune cell composition, function, and metabolic status are strongly influenced by the tumor microenvironment (TME) [6–8]. The complex dynamics of the OSCC microenvironment alter spatiotemporal distribution and effector functions of infiltrating leukocytes to modify/diminish host defense mechanisms in favor of tumor cell survival [9, 10]. In particular, local oxygen depletion leads to the induction of reactive oxygen species (ROS) that promote cancer cell proliferation and drive autophagic/lysosomal loss of stromal caveolin-1 [an inhibitor of transforming growth factor-β (TGF-β) signaling] in cancer-associated fibroblasts (CAFs), resulting in tumor recurrence and metastasis and affecting patient survival [11–14]. Furthermore, the elevated levels of ROS result in detrimental stabilization of hypoxia-inducible factor (HIF)-1α, which activates pro-angiogenic genes including vascular endothelial growth factor (VEGF) [15–17]. HIF-1α acts as a master regulator of oxygen concentration to stimulate hypoxia-adaptive responses in cells. Immune signaling can be altered through the production of onco-metabolites that may further influence the clinical course of OSCC [18]. Consequently, a better understanding of how hypoxic stress, ROS generation, and onco-metabolites alter immune function in the TME is now a priority issue for the OSCC research community. The present article therefore reviews current knowledge of how redox factors alter leukocyte metabolism to promote the immune suppressive microenvironment of hypoxic OSCC.

## Hypoxia and Redox Balance in the Oral Squamous Cell Carcinoma Microenvironment

Hypoxic TME alters local ROS generation and metabolic profile of both constituent tumor cells and infiltrating leukocytes [19–21]. Glycolysis is a metabolic process carried out in cell cytoplasm to generate two ATPs and pyruvates; this pyruvates serves as a fuel for tricarboxylic acid (TCA) cycle and oxidative phosphorylation (OXPHOS) under aerobic conditions [22, 23]. However, under anaerobic conditions, pyruvate is reduced to lactate, and this lactate is secreted into extracellular matrices [24]. The metabolic features of cancer cells are very heterogeneous where OXPHOS and aerobic glycolytic activities are impaired [25].

**Figure 1:**
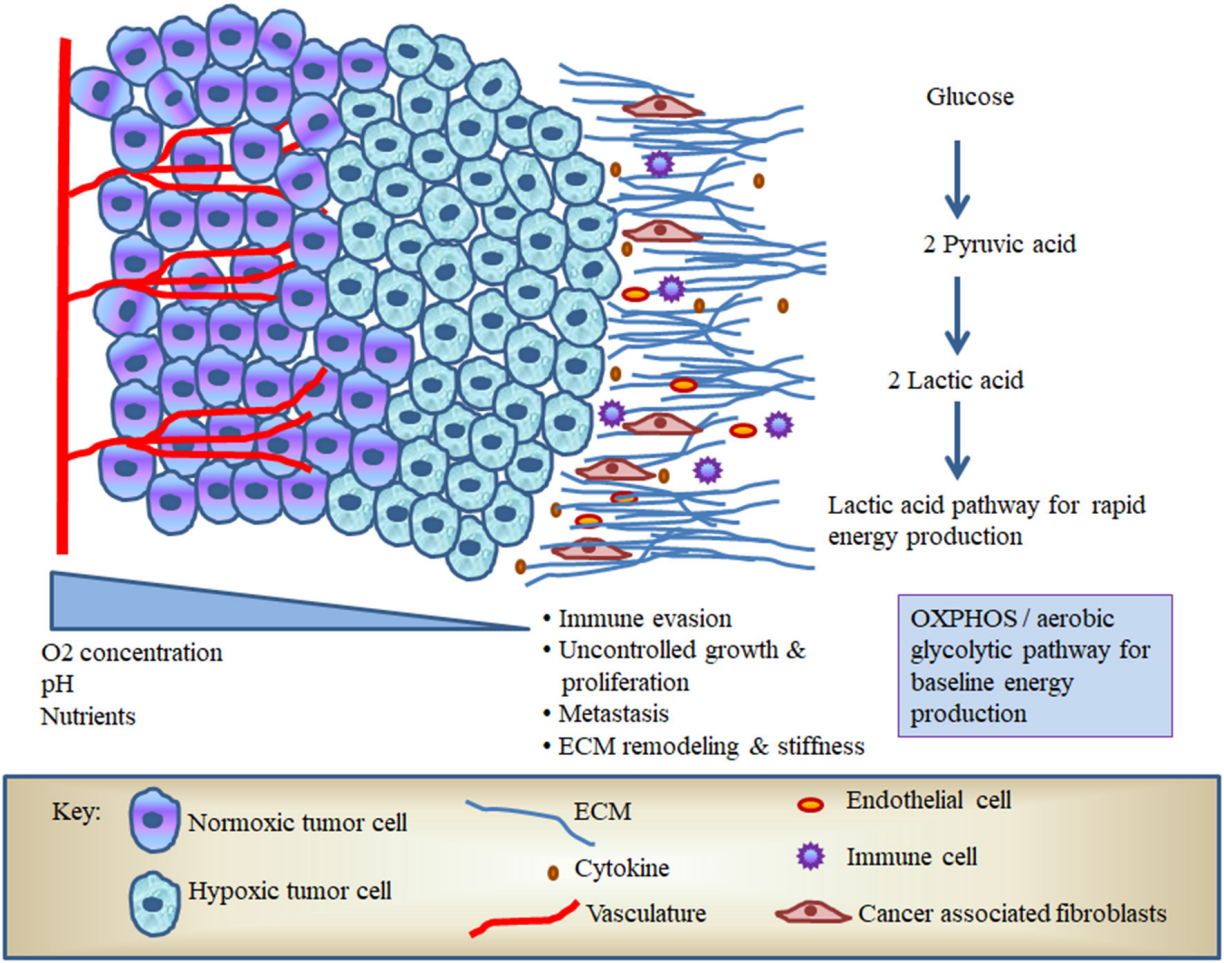
The hypoxic microenvironment of oral squamous cell carcinoma (OSCC).

Intriguingly, cancer cells specifically express pyruvate kinase M (PKM)-2 that oxidizes and generates reduced nicotinamide adenine dinucleotide phosphate (NADPH) to maintain redox buffering; besides, this PKM-2 gene trans-activates HIF-1α target genes, leading to a significant shift in metabolic activity and cancer cell signaling [26, 27]. In solid tumors like OSCC, increased production of ROS, cytokines, and CAFs stimulates the production of pro-angiogenic factors in an attempt to promote neovascularization and enhance survival (Figure 1) [28, 29]. In addition, the cancer cells undergo a metabolic shift from OXPHOS to glycolysis, which produces lactate and increases serum levels of lactate dehydrogenase (LDH), which has been linked with poor survival in patients with OSCC [30]. Tumor cells therefore have the capacity to utilize both OXPHOS and aerobic glycolysis for baseline metabolic activity and rapid energy production *via* the lactate pathway (Figure 1). In this regard, Otto Warburg proposed that due to mitochondrial defects, the predominant metabolism in cancer cells is aerobic glycolysis rather than OXPHOS. Warburg's historic findings were called Warburg effect [31]. However, the metabolic coupling between OSCC cells and associated stromal cells is mainly determined by growth requirements; these effects are called dual/reverse Warburg effect [32, 33]. A previous NMR-based study suggested that OSCC can contravene the Warburg effect and implicated malonate (a competitive inhibitor of succinate) to induce drastic alterations in the TCA cycle that produce more fatty acid for membrane biogenesis in OSCC [34–36]. Among various glycolytic enzymes, alpha-enolase is crucial to produce phosphoenolpyruvate. As the mortality of OSCC is known to be due to metastasis, enolase in particular seems to play a major role in the malignant transformation of dysplastic epithelium in oral pre-cancer through promoting cell surface receptor enolase [37–40]. In addition to altered glucose metabolism, modified amino acid metabolism also occurs in OSCC. The amplified glutamine catabolism creates glutamine scarcity in hypoxic tumor core and leads to a dramatic histone hypermethylation [41]. In order to better understand the effects of hypoxia on OSCC, we will require new immunological paradigms that consider how dysregulation of crucial metabolic pathways can impact on both tumor growth and host leukocyte responses.

## Mitochondrial Homeostasis and Immune Dysfunction in Oral Squamous Cell Carcinoma

In the hypoxic/acidic TME, reduced OXPHOS and electron transport chain (ETC) activity in local immune cells lead to altered mitochondrial membrane potential and impaired generation of ATP [42, 43]. The immune system not only kills cancerous cells but also modifies the TME in three phases—elimination, equilibrium, and escape [44]. The growing and transformed cells can be eradicated by immune response in the elimination phase; however, immune selection and reorganization create an immune resistant environment, namely, the equilibrium phase [45]. Consequently, immune surveillance escapes to kill tumor cells, and tumor cells grow in an uncontrolled manner [46]. In the last phase of cancer immuno-editing (i.e., “escape” phase), cancer cells produce large amounts of “pro-tolerogenic” kynurenine catalyzed by indoleamine 2,3-dioxygenase (IDO) processing of tryptophan [47]. Tryptophan catabolites have affinity to bind aryl hydrocarbon receptors (AhRs) of mitochondria, which persuade mitochondrial dysfunction in T cells and natural killer (NK) cells [48, 49]. Thus, under conditions of acute tryptophan depletion, central mitochondrial metabolic processes and synthesis of NADPH are disrupted such that infiltrating immune cells will undergo apoptosis rather than eradicating the tumor [50].

## Heterotypic Immune Modulation in Hypoxic Oral Squamous Cell Carcinoma

**Figure 2:**
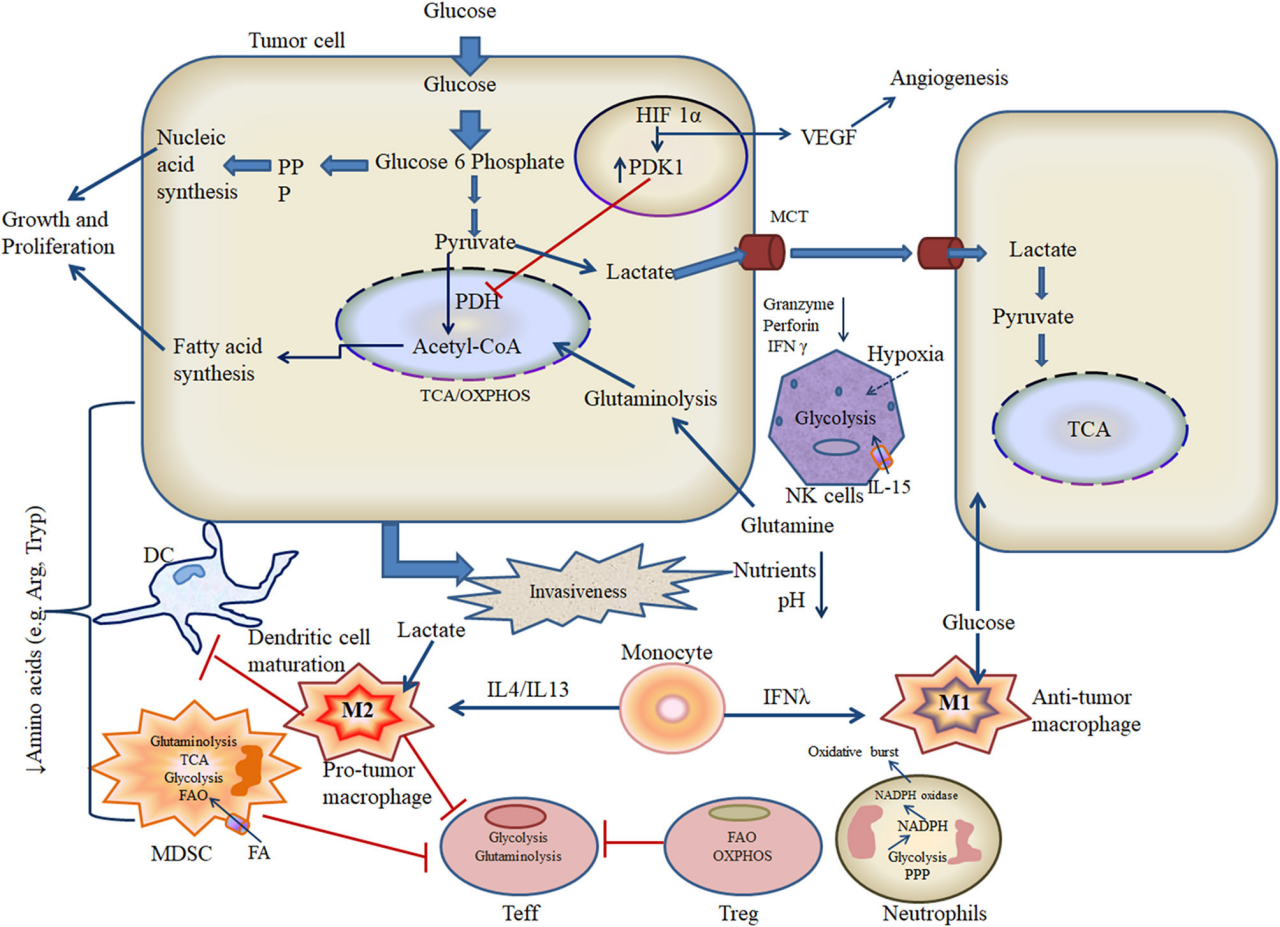
Metabolic crosstalk of tumor and immune cells in the hypoxic microenvironment.

Hypoxic OSCC reprograms cellular metabolism in order to modify the repertoire of infiltrating immune cells toward a more tumor-permissive profile [51]. For example, macrophages located within hypoxic tumors tend to polarize toward an “anti-inflammatory” M2 phenotype, whereas cytotoxic T lymphocytes shift from glycolysis to OXPHOS-based metabolism (Figure 2) [52, 53]. Since essentially all OSCC tumors are subject to hypoxia upon reaching a certain mass, it is important to study how innate and adaptive immune cells alter their metabolism under these conditions in order to fully understand their influence on disease progression.

### Hypoxia “Edits” Immune Signaling

The OSCC microenvironment has two forms of immune responses: innate and adaptive. Innate immune responses are non-specific and instant against pathogens, allergens, and non-self proteins. Phagocytes [myeloid-derived suppressor cells (MDSCs), neutrophils, monocytes, and macrophages] and NK cells are the main cells of innate immunity. Phagocytes engulf the foreign particles and digest through lysosomal enzymes, whereas NK cells kill the foreign bodies using altered major histocompatibility complex I (MHC class I) proteins, perforin, and granzyme-mediated apoptosis [54]. Dendritic cells (DCs) serve as a crucial link between innate and adaptive immune responses under physiological conditions. DCs process antigens and present them to T lymphocytes via MHC class I or II [55]. Adaptive immunity is composed of mainly T and B lymphocytes; B cells are professional antigen-presenting cells (APCs) that can activate T cells in tertiary lymphoid structures, allowing coordination of B and T cell responses in OSCC [56, 57]. In head and neck squamous cell carcinoma (HNSCC), regulatory T cells (T_reg_) situated in the center of the tumor mass have been reported to be more strongly immunosuppressive than circulating T_reg_ [58]). T_reg_ can impede T effector (T_eff_) cell function to reduce antitumor activity and contribute to poor prognosis in multiple types of cancer. Activated CD8+ T_eff_ are dominant antitumor cells that secrete granzymes, perforin, and pro-inflammatory cytokines, such as tumor necrosis factor (TNF) and interferon (IFN)-γ, whereas CD4+ T cells can either inhibit or promote tumor cell activity *via* the specific activities listed here [59, 60].

The macrophage subtypes M_1_ and M_2_ are activated in response to microbial and cancer-derived stimuli, respectively [61]. M_1_ polarization of macrophages is induced by T helper type 1 (T_H_1) cytokines such as IFN-γ and signaling through signal transducer and activator of transcription 1 (STAT1), whereas M_2_ polarization is promoted by T helper type 2 (T_H_2) cytokines such as IL-4 and IL-13 that trigger the STAT6 pathway [62]. Functionally, M_1_ macrophages produce pro-inflammatory cytokines, ROS and reactive nitrogen species (RNS), while mediating antigen presentation *via* MHC class II molecules. M_1_ macrophages also actively phagocytose pathogens and are considered to suppress tumor development [63]. In contrast, M_2_ macrophages are activated by cytokines including IL-4/IL-13, IL-10, TGF-β, and glucocorticoids that promote secretion of anti-inflammatory mediators. Further, M2 macrophages inhibit the lytic activity of CD8+ cytotoxic T cells [55]. Despite their opposing roles, both M1 and M2 macrophages can coexist within the same tumor.

Tumor cells can secrete IL-10, colony-stimulating factor (CSF)-1, and various chemokines [C-C motif chemokine ligand (CCL)-2, CCL-18, CCL-17, and C-X-C motif chemokine ligand (CXCL)-4] that appear to favor M_2_ polarization [64]. In addition to cytokine expression, hypoxic tumors can further direct macrophage phenotypes and responses via release of exosomes loaded with soluble factors and suppressive micro RNA [65]. In human HNSCC, the acidic TME has been reported to promote HIF-1α activation and tumor-associated macrophage (TAM) expression of M_2_-specific markers CSF1R and CD163, as well as driving concomitant production of arginase and VEGF [66, 67]. NK cells have the capacity to kill tumor cells and activate antitumor T cell responses by secreting IFN-λ and cytotoxic molecules such as granzyme and perforin, but these activities can be severely restricted by the concomitant presence of MDSCs [68, 69]. MDSCs are known for their immune suppressive activity for both innate and adaptive immunity. The two subtypes of MDSCs are monocytic (M-MDSCs) and polymorphonuclear (PMN-MDSCs), which have variable capacities to inhibit the function of activated CD8+ T cells [70, 71]. MDSCs are thought to suppress T cell responses by expressing a range of inhibitory factors including arginase, inducible nitric oxide synthase (iNOS), TGF-β, IL-10, cyclooxygenase (COX)-2, and IDO [72]. HIF-1α appears to play a key role in this immunosuppressive process by driving the expression of cytokines that promote MDSC infiltration of the tumor mass [73]. In addition, granulocyte MDSCs (G-MDSCs) are classified as T cell-suppressive neutrophils because of similar morphology and cell surface markers as mature neutrophils [74, 75]. Neutrophils migrate toward and infiltrate tumors under the influence of potent chemokines such as IL-8, after which these cells appear to enhance tumor proliferation and are correlated with poor survival in solid cancers [76, 77]. Several clinical observations indicate that neutrophil activity is further modulated under the influence of the TME to assist cancer development [78]. However, tumor-associated neutrophils (TANs) may exert a dual role, since these cells appear capable of promoting either CD8+ T cell activity or tumor progression, depending on the prevailing level of TGF-β within the TME [79].

Likewise, with the polarization of TAMs, TANs exhibit two polarization phenotypes, i.e., N1 (antitumor neutrophils) and N2 (pro-tumor neutrophils), where TGF-β signaling plays a vital role [80]. Obstruction of TGF-β signaling or type I IFNs activates N1 phenotype with accretion of TNF-α and type 1 IFN, whereas augmentation in TGF-β signaling leads to N2 phenotype with high levels of neutrophil elastase (NE) and arginase in oral cancers [81]. Immature DCs are activated by pathogen-associated molecular patterns such as toll-like receptor (TLR) ligands, thereafter migrating to lymphoid organs and presenting antigen to T cells in the context of MHC [82]. In this process, phosphoinositide 3-kinase (PI3K)/Akt signaling pathway regulates the metabolic switch through inhibiting AMP-activated protein kinase and promotes glycolysis [83]. While adenosine signaling limits DC activation, ATP detection by P2YR and P2XR promotes DC migration and IL-1 secretion, respectively [84]. Hypoxic TME not only alters the innate immune signaling but also modifies adaptive immune signaling. Likewise, under hypoxic conditions, B cell caspase signaling is activated and kinase complex mammalian target of rapamycin complex 1 (mTORC1) pathway is reduced, leading to cell death via apoptosis [85]. B cells also secrete IL-10 under hypoxic stress; however, in-depth molecular pathway is not well-characterized [86]. The role of HIF-1α transcription factor in tumor-infiltrating T cells remains unclear [87]. However, in cancer cells, HIF-1α interaction with hypoxia response element (HRE) in the programmed cell death ligand 1 (PD-L1) promoter can trigger rapid expression of this immune checkpoint molecule, which is also capable of signaling more effectively in the lactate-rich TME [59, 88]. PD-L1 ligation of programmed cell death protein 1 (PD-1) on T_eff_ can inhibit T cell receptor (TCR) signaling and attenuates the PI3K/Akt and Ras/mitogen activated protein kinase (MEK)/extracellular signal-regulated kinase (ERK) pathways to restrict antitumor responses [89, 90]. Accordingly, antibodies targeting the PD-1: PD-L1 axis and other immune checkpoints have the ability to restore glucose levels in the hypoxic TME and have proven highly effective in the treatment of OSCC [91–93]. In particular, abnormal metabolic processes within cancer cells can generate neo-antigens that are presented by MHC class I molecules on the cell surface of antigen-presenting cells for recognition by CD8+ cytotoxic T cells [94].

### Hypoxia Modifies the Resting Metabolic Status of Immune Cells

The resting metabolic status and associated effector functions of local immune cells play vital roles in determining the nature of host antitumor responses. In particular, glucose transport regulates pyruvate flow into the TCA cycle and is essentially “rate-limiting” for host immunity, since leukocytes typically require rapid energy generation in order to achieve full activation. The hypoxic TME is a key driver of M_2_ polarization in infiltrating macrophages likely via the expression of specific cytokine signals that activate nuclear factor (NF)-κB, although the underlying mechanism has yet to be precisely defined in OSCC [95, 96]. Like other myeloid lineage cells, macrophage mitochondria can generate both superoxide and NO, which react to form the powerful oxidant peroxynitrite, which is highly toxic to cancer cells [97, 98]. While some investigators have reported that mitochondrial ROS (mROS) stimulate macrophage expression of pro-inflammatory cytokines, other researchers have instead observed the induction of an anti-inflammatory phenotype; hence, further study is required to fully understand these events [99–102]. The glycolytic reprogramming of TAM is regulated by oxygen sensors including prolyl-hydroxylases (PHDs) and is accompanied by proton pumping and acidification of M2 macrophages that subsequently impair antitumor responses [103]. Similarly, while resting NK cells typically utilize OXPHOS, exposure to high doses of tissue damage-associated cytokine IL-15 stimulates conversion to glycolytic activity [104]. For tumor-infiltrating neutrophils, the principal metabolic pathways employed are aerobic glycolysis and the pentose phosphate pathway (PPP), which support chemotaxis and microbicidal activities, respectively (Figure 2) [105]. Metabolic shift toward PPP is also required for formation of neutrophil extracellular traps, which envelope and attach to the circulating cancer cells and expedite metastasis to distant sites [106]. Tumor-associated MDSCs predominantly utilize fatty acid (FA)-β oxidation (FAO) and thus display high rates of oxygen consumption (Figure 2) [107]. In the hypoxic TME, MDSCs display potent immunosuppressive activity, which depends on the endoplasmic reticulum (ER) stress response transcription factor CCAAT-enhancer-binding protein homologous protein (CHOP) [108]. DCs are the critical components of the immune system against cancer as they have robust antigen-presenting ability to educate T cells [109, 110]. Upon microbial stimulation, DCs typically shift from OXPHOS to glycolysis; however, in TME, DCs promote immune suppression through galectin-1 [111]. In this regard, how metabolic profiles influence DC function and tumor progression *in vivo* is not yet well-defined [56, 112]. Activated B cells can secrete antibodies that can bind and induce tumor cell killing, but these processes can be strongly influenced by mitochondrial generation of ROS and heme synthesis. While the presence of CD20+ B cells within the TME indicates a good prognosis in lung cancer, gastric cancer, and melanoma, the role played by B cells in HNSCC has yet to be fully investigated. Variable hypoxia across the developing OSCC tumor is thought to alter tissue distribution of local B cells, which generate immune complexes and produce cytokines that then modify myeloid cell function to assist tumor progression [113]. B cells utilize glycolytic metabolism during early development in the bone marrow. Later survival, maturation, and functional activity of B cells are instead regulated by HIF-1α and depend on glucose transporters and phosphofructokinase. The oncogenic Myc expression in B cells hindered the oxidation of acetyl-CoA in TCA cycle as it persuades lactate dehydrogenase for conversion of pyruvate (glycolysis intermediate) to lactate [114]. Naive T cells employ OXPHOS and fatty acid metabolism before shifting glycolysis in order to support T_eff_ functions [112]. Memory T cells also depend on OXPHOS for energy generation in the resting state, whereas T_reg_ favor fatty acid oxidation. It is noteworthy that while glycolytic metabolism predominates among T_eff_ and T_reg_, both populations have been observed to maintain OXPHOS within the TME (Figure 2), which may contribute to detrimental cancer-associated inflammation, further mutation, and eventual metastasis [115, 116]. Metabolic alterations in the T cell pool may also impede the differentiation of T_eff_ while increasing the generation of T_reg_ and “exhausted” populations, thus further supporting cancer evasion of host immunity [50].

### Hypoxic Oral Squamous Cell Carcinoma Produces Immunosuppressive Onco-Metabolites

The metabolic products of cancer cells (onco-metabolites) are intimately linked with the control of the immune cell function [117]. For example, the microenvironment of OSCC is characterized by hypoxia, low pH, and elevated lactate levels, which disturb ETC operation and leads to deposition of citrate and succinate [21]. Citrate is converted into acetyl-CoA and utilized in several biosynthetic pathways. Oxidation of succinate produces ROS and promotes HIF-1α activation. Hypoxia-generated lactate also drives macrophage differentiation toward an M2 phenotype [118]. TAMs are unable to utilize extracellular arginine due to rapid enzymatic breakdown by arginase and must instead use extracellular glutamine to produce this “semi-essential” amino acid [119]. TANs also produce high levels of arginase to disrupt TCR signaling. Increased lactate concentration in the hypoxic TME favors decreased NK cell expression of granzyme/perforin and NKp46, leading to reduced anticancer cytolytic activity (Figure 2) [120]. The nuclear factor of activated T cells (NFAT) has also been implicated in downregulation of NK cell activity *via* an increase in cancer-associated lactate dehydrogenase expression [121]. Other hypoxia-induced onco-metabolites such as adenosine and lactic acid have previously been reported to impair DC activation (Figure 2) [122]. The tumor-associated dendritic cells (TADCs) stimulate arginase, which then depletes arginine in the extracellular matrix (ECM) and arginine scarcity impairs CD8+ T cell responses [123]. Some stable onco-metabolites (like kynurenine and quinolinate) along with specific cytokine milieu promote AhR signaling in non-functional T_reg_, Foxp3+-induced T_reg_ (iT_reg_), and T_H_17 cells. These signaling pathways further de-differentiate T_reg_, iT_reg_, and T_H_17 cells into functional iT_reg_ and endorse immune tolerance [124, 125]. IDO contributes to the tolerogenic ability of DCs to inhibit T_eff_ functions and promotes T_reg_ activity [126, 127]. The by-products (highly reactive aldehyde) of anomalous lipid peroxidation (triggered by ROS) create ER stress on TADCs and lower antitumor responses [128]. In addition, acylcarnitine and 2-hydroxyglutarate (2-HG) have been identified as a prominent onco-metabolite in HNSCC [129]. This 2-HG skews T_H_17 polarization and alters T_reg_ metabolism by promoting the OXPHOS and destabilizing HIF-1α [130, 131].

## Immune-Tumor Metabolic Switch Under Hypoxia

Tumor hypoxia is characterized by local tissue acidification and nutrient depletion, thereby creating metabolic competition and generating active biomolecules that influence cancer cell interactions with host leukocytes. Competition for key metabolites along with cholesterol esterification, release of adenosine, and expression of prostaglandin E2 inhibits effector T cells [132]. Hypoxic OSCC modifies pro- and antitumoral γδ T cell populations *via* exosomes [11]. OSCC can also express a range of different TLRs, with high levels of TLR-2 and TLR-4 correlating with tumor progression and chemoresistance, respectively. HIF-1 can deregulate *TLR3* and *TLR4* in OSCC cell lines under hypoxia stress, leading to potent effects on tumor cell survival, proliferation, and metastatic potential [133].

## Concluding Remarks and Future Directions

Hypoxia-related metabolic stress inhibits the activity of host immune cells to support oncogenic transformation and inflammation in OSCC. The hypoxia signaling in immune cells not only alters the glycolysis but also modifies other metabolic pathways like amino acid, FAO, PPP, and TCA, resulting in onco-metabolite production. Further, these onco-metabolites disturb the redox balance, mitochondrial function, and ATP production through aerobic glycolysis in OSCC.

In future studies, it will be important to elucidate the correlation between spatial distributions of immune cells in hypoxic/non-hypoxic OSCC. Novel and advanced therapeutic approaches like interfering with HIF-1α signaling in immune cells through antisense or small interfering RNA, modulating the metabolic status of immune cells through gene editing technology [clusters of regularly interspaced short palindromic repeats–caspase 9 (CRISPR-Cas9)], and designing new smart oxygen-sensitive chimeric antigen receptor (CAR) T cell may provide new insight to overcome the challenges associated with hypoxic OSCC in the future.

## Author Contributions

AC conceptualized, conceived, and wrote the manuscript. SB conceptualized and organized the review. NA guided and revised the manuscript. VR revised and approved the manuscript. DM guided, revised and approved the manuscript. GM guided to write, organized and revised the manuscript. All authors contributed to the article and approved the submitted version.

## Conflict of Interest

The authors declare that the research was conducted in the absence of any commercial or financial relationships that could be construed as a potential conflict of interest.
